# Association of Trimethylamine N-Oxide (TMAO) with the Clinical Severity of Hidradenitis Suppurativa (Acne Inversa)

**DOI:** 10.3390/nu13061997

**Published:** 2021-06-10

**Authors:** Luigi Barrea, Giovanna Muscogiuri, Gabriella Pugliese, Giulia de Alteriis, Maria Maisto, Marianna Donnarumma, Gian Carlo Tenore, Annamaria Colao, Gabriella Fabbrocini, Silvia Savastano

**Affiliations:** 1 Dipartimento di Scienze Umanistiche, Università Telematica Pegaso, Centro Direzionale, Via Porzio, isola F2, 80143 Napoli, Italy; luigi.barrea@unipegaso.it; 2 Centro Italiano per la cura e il Benessere del Paziente con Obesità (C.I.B.O.), Endocrinology Unit, Department of Clinical Medicine and Surgery, University Federico II, Via Sergio Pansini 5, 80131 Naples, Italy; giovanna.muscogiuri@gmail.com (G.M.); robiniapugliese@gmail.com (G.P.); colao@unina.it (A.C.); 3 Unit of Endocrinology, Dipartimento di Medicina Clinica e Chirurgia, Federico II University Medical School of Naples, Via Sergio Pansini 5, 80131 Naples, Italy; dealteriisgiulia@gmail.com; 4 Cattedra Unesco Educazione alla Salute E Allo Sviluppo Sostenibile, University Federico II, 80131 Naples, Italy; 5 Department of Pharmacy, University of Naples Federico II, Via Domenico Montesano, 49, 80131 Naples, Italy; maria.maisto@unina.it (M.M.); giancarlo.tenore@unina.it (G.C.T.); 6 Section of Dermatology, Department of Clinical Medicine and Surgery, University of Naples Federico II, 80131 Naples, Italy; mavabe@hotmail.it (M.D.); gafabbro@unina.it (G.F.)

**Keywords:** trimethylamine n-oxide (TMAO), acne inversa, hidradenitis suppurativa, Mediterranean diet, phase angle, nutritionist

## Abstract

In this case-control, cross-sectional, observational study, we evaluated circulating trimethylamine n-oxide (TMAO) levels, a gut-derived metabolite associated with inflammation and cardiometabolic risk, in patients with hidradenitis suppurativa (HS), a highly disabling inflammatory skin disease associated with an elevated prevalence of comorbidities, especially cardiovascular and metabolic diseases. In this study, we enrolled 35 naive-treatment patients with HS and 35 controls, matched for sex, age, and body mass index (BMI). HS Sartorius score was 49.0 (33.0–75.0), while according to the Harley system 12 and 23 patients presented grade 1 and grade 2 severity, respectively. HS patients had a lower adherence to the Mediterranean diet (MD) (*p* = 0.002), lower phase angle (PhA) (*p* < 0.001), and higher circulating TMAO levels (*p* < 0.001) than the control group. HS patients with grade 2 rather than grade 1 of Harley grade severity showed a higher BMI (*p* = 0.007), waist circumference (*p* = 0.016), total energy intake (*p* = 0.005), and lower PhA (*p* < 0.001) and adherence to the MD (*p* = 0.003). Of interest, patients with Hurley grade 2 of severity exhibited higher circulating TMAO levels (*p* < 0.001) compared to grade 1. Circulating TMAO levels showed a positive correlation with HS Sartorius score even after adjustment for confounding covariates, including BMI, waist circumference, adherence to the MD, total energy intake, and PhA (r = 0.570, *p* = 0.001). Using a linear regression model, circulating TMAO levels and PhA were the main predictors of the clinical severity of HS.

## 1. Introduction

Trimethyilamine N-oxide (TMAO) is a gut-derived metabolite that depends on the function of the intestinal barrier and is associated with an increased risk of metabolic syndrome, cardiovascular disease, and mortality [1]. Besides the gut microbiota, circulating TMAO levels are determined by many factors, such as age, gender, and dietary nutrients [2]. Among gut-derived metabolites, TMAO has been associated with oxidative stress and low-grade chronic inflammation through the dysregulation of specific molecular inflammatory pathways [3]. Increased circulating TMAO levels have been frequently associated with non-alcoholic fatty liver disease, and chronic kidney diseases, and inflammatory bowel disease [4]. TMAO is considered a marker of bacterial translocation, gastrointestinal symptoms, and systemic inflammatory profile [5,6].

Hidradenitis suppurativa (HS) is an auto-inflammatory and highly disabling skin disease, commonly presenting with painful subcutaneous nodules in intertriginous anatomical sites of the body, such as the axillary, inguinal and perianal areas [7]. HS onset generally occurs after puberty, with the highest incidence in the third decade of life. Although it affects both sexes, HS is mainly diffused among women [8]. The exact prevalence of this disease is still undefined, but it has been reported as having a prevalence of 1% in the general population in Europe [9,10], which can reach up to 4% in young adult women [11], and between 0.05% and 0.20% in American epidemiological studies [12,13]. To date, clinical skin lesions and their chronicity play an important role in establishing the diagnosis of patients with HS, but reliable and accurate diagnostic tests are still lacking [14].

Although its pathophysiology up to now is not well understood, HS is considered a systemic disease, due to its association with an elevated prevalence of comorbidities, especially cardiovascular and metabolic diseases, commonly characterized by a state of low-grade chronic inflammation [15]. However, HS could also be a multifactorial condition, in which genetic and/or environmental factors, including altered microbial composition (termed microbiota dybiosis), nutrition, and obesity, are strictly associated with its pathogenesis [16,17,18,19]. Gastrointestinal dysbiosis might contribute to HS pathogenesis through an increased intestinal permeability and the disregulation of multiple pathways that include the production of metabolites triggering inflammation in predisposed individuals [19,20]. Deckers IE et al. in a multicenter, cross-sectional study including a total of 1076 HS patients reported that the prevalence of inflammatory bowel diseases is 4–8 times higher in the HS cohort compared with the general population, thus confirming the association between these two diseases [21]. The association between the HS and inflammatory bowel diseases suggests that patients with HS may have a common disruption in their immune milieu and gut microbial community [22], in the context of the skin gut axis [23,24].

The influence of nutrition and obesity in patients with HS is supported by emerging evidence [25]. In particular, we previously reported a significant association between the clinical severity of HS and the degree of adherence to the Mediterranean diet (MD), a nutritional model based on the eating habits and traditional lifestyle typical of the Mediterranean countries, which is well known to exert anti-inflammatory and immune-modulating effects [26]. On the other hand, it is also known that nutrition influences the composition of the gut microbiome, which, in turn, affects a wide range of hormonal and metabolic processes [27].

Obesity is an important risk factor for HS patients, with rates of obesity that vary from 12% to 88% among them [28]. Patients with HS presented a higher body mass index (BMI) [28], larger waist circumference [29,30], and also a higher percentage of body fat than healthy controls, independent of their BMI [31,32]. In addition, we have previously reported that phase angle (PhA), a bioelectrical impedance analysis (BIA)-derived measure associated with inflammatory status [33,34,35], and a prognostic index for chronic inflammatory processes, was significantly lower in HS patients, similarly to other skin inflammatory diseases, such as psoriasis or acne [26,36,37].

Since the assessment of TMAO as an indirect marker of gut dysbiosis in patients with HS has not been established in previous studies and no data are available in the literature, the mechanisms linking circulating TMAO levels to the clinical severity of HS are not clear, and represent a topic of great interest for endocrinologists, nutritionists, and dermatologists.

The aim of the present study was to investigate the possible difference in circulating TMAO levels in a sample of naive-treatment patients with HS compared to a control group matched for sex, age, and BMI. In addition, we aimed to assess the possible association of circulating TMAO levels with the clinical severity of HS according to possible confounding effects of the nutritional status.

## 2. Materials and Methods

### 2.1. Design and Setting

This case-control, cross-sectional, observational study was conducted in patients attending the Department of Clinical Medicine and Surgery, Federico II University Hospital of Naples (Italy), from January 2017 to May 2018. Healthy subjects were recruited during the Obesity, Programs of nutrition, Education, Research, and Assessment of the best treatment (OPERA) prevention project [38]. This monocentric study, approved by the Federico II Ethical Committee with protocol number n. 201/15, was registered at clinicaltrials.gov as NCT03683238. The study was carried out in accordance with the Declaration of Helsinki. The aim of the research was clearly explained and written informed consent was obtained from all participants. The study was conducted without support from the pharmaceutical industry.

### 2.2. Population Study

The study included 70 Caucasian participants; 35 HS patients, and 35 subjects without HS, used as a control group. Controls were matched to HS patients by age, gender, and BMI. Patients with HS and control subjects came from the same geographical area around the Naples metropolitan area, Campania, Italy.

Patients with HS were eligible for this study according to the following criteria:

1. Treatment-naive adult patients;
2. HS diagnosed ≥6 months before the study initiation and without medical therapy for at least 3 months;
3. All three diagnostic criteria for HS had to be met: presence of typical lesions, anatomical sites involved in typical areas, and an evolving disease course with relapse and chronicity;
4. Clinical severity: mild to moderate HS (Hurley stage I or II);

The exclusion criteria for both HS patients and controls are listed below:

1. Symptoms or signs of androgen excess or endocrine disorders;
2. Menopausal females or current continued use of hormonal contraceptives, pregnancy, or lactation in the past 6 months;
3. Occasional or current use of systemic treatments (including biologics, cyclosporine A, rifampicin–moxifloxacin–metronidazole, clindamycin–rifampicin, dapsone, ertapenem, tetracycline, acitretin, and isotretinoin) or other drugs for HS, including topical antibiotics;
4. Hypocaloric diet, other specific dietary patterns, including vegetarian diet or ketogenic diet, or subjects who supplemented their diet with antioxidants, vitamins, minerals, or probiotics in the last three months;
5. Clinical conditions or use of drugs that could influence fluid balance, including liver or renal failure (estimated glomerular filtration rate (eGFR) < 90 mL/min/1.73 m^2^), cancer, and other chronic or acute diseases, were based on a complete medical examination and laboratory tests;
6. Patients with any other active skin disease (e.g., psoriasis and acne) that could interfere with the assessment of HS;
7. Patients with type 2 diabetes (glycated haemoglobin (HbA1c) ≥ 6.5% (≥48 mmol/mol) or two confirmations of fasting glucose ≥ 126 mg/dL, in according with the American Diabetes Association criteria);
8. Current use of hypoglycemic, anti-inflammatory, or hypolipidemic drugs;
9. Individuals with implanted pacemakers or defibrillators (for the theoretical possibility of interference with the BIA-device activity);
10. History of any clinical condition that, in the opinion of the nutritionist, endocrinologist or dermatologist, could put the patient at risk if they participated in this study.

### 2.3. Sample Size Justification

The sample size was calculated by the difference of means ± standard deviation (SD) of circulating TMAO levels in the two independent study groups, patients with HS and controls (6.22 ± 4.18 and 4.03 ± 2.55 µM, respectively). The number of individuals enrolled was found to be 68 subjects, 34 per group, with an effect size of 0.95 with a type I error of 0.10, and a power of 70%, as previously reported [39,40]. The calculation of sample size and power were performed using Sample Size Calculator Clinical Calc (https://clincalc.com/stats/samplesize.aspx, accessed on 21 March 2021).

### 2.4. Lifestyle Informations

Physical activity levels and smoking habits were assessed in all study participants through a standard questionnaire, as previously reported [41,42]. Active subjects were classified as 1, while inactive individuals as 0. Likewise, current smokers (when smoking at least one cigarette per day) were classified as 1, while non-current smokers as 0, as we reported earlier [43,44].

### 2.5. Anthropometric Measurements

A certified clinician performed anthropometric measurements on all participants. All subjects wore light clothing and were without shoes when all anthropometric measuring was done, as shown in previous studies. The participants were assessed after an overnight fast of 12 h between 8 am and 10 am. In detail, height and weight were assessed with a calibrated balance with stadiometer (Seca 711; Seca, Hamburg, Germany to the nearest 1 cm and 0.1 kg for height and weight, respectively), as previously reported [45,46,47]. In accordance with the World Health Organization (WHO)’s criteria [48], BMI was calculated by weight (kg) and height squared (m^2^). Subjects were classified into four BMI classes: grade II obesity, grade I obesity, overweight, and normal weight (BMI: 35.0–39.9 kg/m^2^, 30.0–34.9 kg/m^2^, 25.0–29.9 kg/m^2^, and 18.5–24.9 kg/m^2^; respectively). According to the National Center for Health Statistics (NCHS), waist circumference was assessed with a non-stretchable measuring tape to the closest 0.1 cm at the narrowest point. In grade I or grade II obesity, where no narrowest point of waist circumference was visible, it was taken at the umbilical level using a non-stretchable measuring tape to the nearest 0.1 cm [49].

### 2.6. Determination of Circulating TMAO Levels

Samples for determination of serum circulating TMAO levels were stored at −80 °C, as it has been shown that under these conditions TMAO remains stable for years [50]. Serum circulating TMAO levels were measured using the method described by Beale and Airs [51], as reported in our previous study [52,53,54]. The chromatographic separation was carried out with a guard column (HILIC), in combination with a Luna HILIC column (150 mm× 3 mm, 5 µm particles), both supplied by Phenomenex (Torrance, CA, USA).

### 2.7. Nutritional Assessments: Adherence to the MD and Total Energy Intake

Using a Prevención con Dieta Mediterránea (PREDIMED) questionnaire, a brief validated questionnaire of 14 items [55], we assessed participants’ adherence to the MD. This questionnaire was administered by a certified clinical nutritionist during a face-to-face interview, and had already been used in previous research [56,57,58]. The PREDIMED score (ranged from 0 to 14 points) was calculated by assigning a score of one (affirmative answer to the question) or zero (negative answer to the question). Participants were classified into three PREDIMED categories, according to the PREDIMED score obtained as follows: low adherence, average adherence, and high adherence to the MD (0–5, 6–9, and score ≥ 10, respectively) [55].

The total energy intake was obtained by a face-to-face interview, as previously reported [43,59]. Seven-day food records were used to collect dietary data and a photographic food atlas of known portion sizes was used to illustrate and quantify foods and drinks to participants to ensure accurate completion of the seven-day food records [60]. Data of the 7-day food records were processed using a commercial software (Terapia Alimentare Dietosystem DS-Medica, http://www.dsmedica.info, accessed on 21 March 2021). This software was able to calculate the total energy intake, expressed in kcal.

### 2.8. Phase Angle

According to the European Society for Clinical Nutrition and Metabolism (ESPEN) [61], body composition was assessed by BIA phase-sensitive system (BIA 101, 800 µA current at a single frequency of 50 kHz, RJL Akern Bioresearch, Florence, Italy) [62]. The BIA exam was performed by the same certified clinical nutritionist and using the same BIA-device to avoid inter-device and inter-observer variability, as previously reported [33,63]. To practice the BIA examination, we used BIATRODES electrodes (Akern Srl, Florence, Italy) based on Kushner’s guidelines [64]. In detail, subjects were asked to remove their shoes and socks, and the contact areas on the ipsilateral foot and hand were scrubbed with alcohol immediately before applying the electrodes. PhA (°, degrees) was obtained from the BIA-device by the formula: reactance (Xc)/resistance (R)*(180/π). All female participants were assessed during the follicular phase of their menstrual cycle to avoid interference with the BIA-device values.

### 2.9. Classification and Severity Assessment of HS

As a gold standard is still lacking, the classification and assessment of the clinical severity of HS was assessed using two scoring systems: Sartorius HS score and Hurley stages [65,66]. In detail, the clinical classification system based on the Sartorius HS score is a validated measure of HS activity and provides the counting of single fistulas and nodules within seven anatomical regions [65]. The Hurley system describes three distinct clinical stages as follows: Stage I: single or multiple abscess formation, without sinus tracts and cicatrization; stage II: single or multiple recurrent abscesses with tract formation and cicatrization, widely separated lesions; diffuse lesions and multiple interconnected sinus tracts and abscesses across the entire area are classified as stage III. In this study, we only enrolled patients with Hurley stage 1 and 2 [67]. Two independent dermatologists evaluated the clinical severity of HS, and they were blinded to the design of the study to prevent avoidable biases.

### 2.10. Statistical Analysis

Data were collected and analyzed using the MedCalc^®^ package (MedCalc® version 16.8.4; MedCalc Software Ltd., Ostend, Belgium). The data distribution was evaluated by Kolmogorov–Smirnov test and the abnormal data were normalized by logarithm. Skewed variables were converted into figures and tables. Results have been described as mean ± SD or percentage/number.

Differences between patients with HS and the control group were analyzed by Student’s paired *t*-test, while the differences among the three groups were evaluated by ANOVA followed by the Bonferroni *post-hoc* test. The chi square (χ^2^) test was used to evaluate the differences in frequency distribution. The correlations between study variables were performed using Pearson *r* correlation coefficients, which were estimated after adjusting for confounding covariates, including BMI, waist circumference, PREDIMED score, total energy intake, and PhA. Proportional Odds Ratio (OR) models, 95% Interval Confidence (IC), and R^2^, were used to assess the associations between the Hurley system and study parameters. A linear regression model was performed for the evaluation of the association between HS Sartorius score (as a dependent variable) and BMI, waist circumference, circulating TMAO levels, PREDIMED score, total energy intake, and PhA as independent variables. Tolerance and variance inflation factor were calculated to determine the presence of collinearity.

## 3. Results

The study population consisted of 70 total participants, 48 females (68.6%) and 22 males (31.4%), aged 18–49 years, and BMIs ranged from 19.2 to 39.9 kg/m^2^. All participants completed the study protocol, including nutritional and dermatological assessments. In detail, the nutritionist evaluated both patients with HS and controls with anthropometric and nutritional assessments, BIA measurements, and blood sampling for the assessment of circulating TMAO levels. Subsequently, the dermatologist ruled out HS in control subjects, in which the clinical severity indices of HS were zero. In HS patients, the Sartorius score was 49.0 (33.0–75.0). According to the Harley system, 12 and 23 patients presented grade 1 and grade 2 of severity, respectively.

Table 1 reported the differences in lifestyle habits, anthropometric measurements, nutritional status, and BIA parameters in HS patients and controls. The two groups did not show statistically significant differences in smoking habits (*p* = 1.00), physical activity (*p* = 0.807), or total energy intake (*p* = 0.193). However, HS patients had a lower adherence to the MD (*p* = 0.002) and lower PhAs (*p* < 0.001) than the control group.

**Figure 1:**
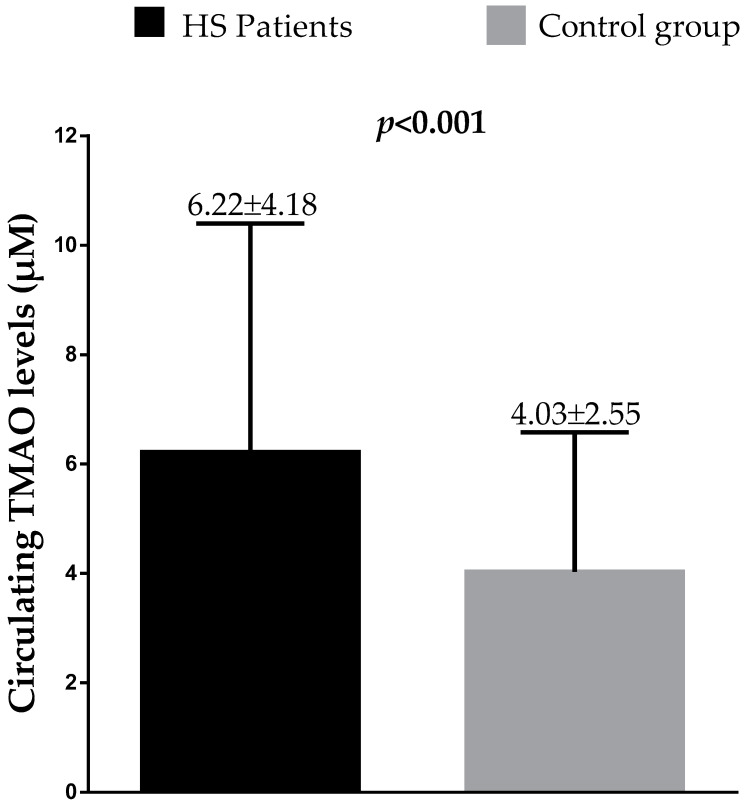
Difference in circulating TMAO levels between HS patients and control group. Difference was analyzed by Student’s paired *t*-test. Results were expressed as mean ± SD. A *p* value in bold type denotes a significant difference (*p* < 0.05). HS, hidradenitis suppurativa; TMAO, trimethylamine n-oxide.

Figure 1 reports the difference in circulating TMAO levels between HS patients and the control group. HS patients had higher circulating TMAO levels compared to controls (*p* < 0.001).

Table 2 reports the demographic characteristics, anthropometric measurements, nutritional assessments, and BIA parameters of HS patients grouped based on the Hurley system. Compared to HS patients with grade 1 of severity, grade 2 patients were more frequently smokers (*p* = 0.001), presented higher BMI (*p* = 0.007) and waist circumference values (*p* = 0.016), and lower PhA (*p* < 0.001). In addition, HS patients with higher clinical severity were less adherent to the MD (*p* = 0.003) and had a higher total energy intake (*p* = 0.005), as shown in Table 2.

**Figure 2:**
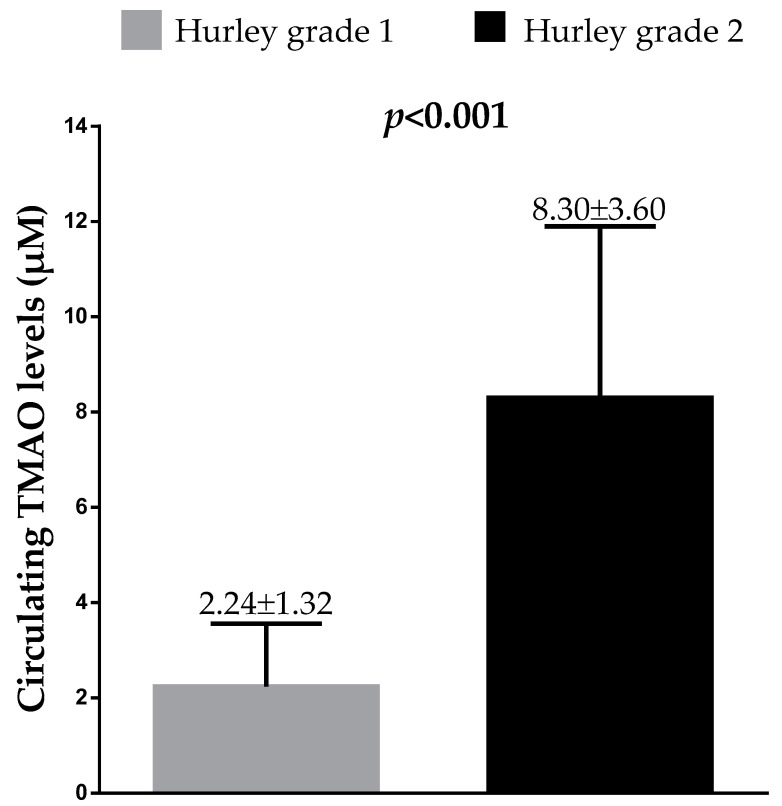
Difference in circulating TMAO levels in HS patients between Hurley grade 1 vs. Hurley grade 2. Difference was analyzed by Student’s impaired *t*-test. Results were expressed as mean ± SD. A *p* value in bold type denotes a significant difference (*p* < 0.05). TMAO, trimethylamine n-oxide.

Figure 2 shows the difference in circulating TMAO levels between HS patients with Hurley grade 1 vs. Hurley grade 2. As shown in Figure 2, patients with the highest clinical severity of the disease (Hurley grade 2) exhibited the highest circulating TMAO levels (*p* < 0.001).

### Correlation Analysis

Correlation analysis between HS Sartorius score with demographic characteristics, anthropometric measurements, nutritional assessments, and BIA parameters in patients with HS are summarized in Table 3. Except for age and Xc, the clinical severity of HS showed a significant correlation with all other parameters evaluated in this study.

**Figure 3:**
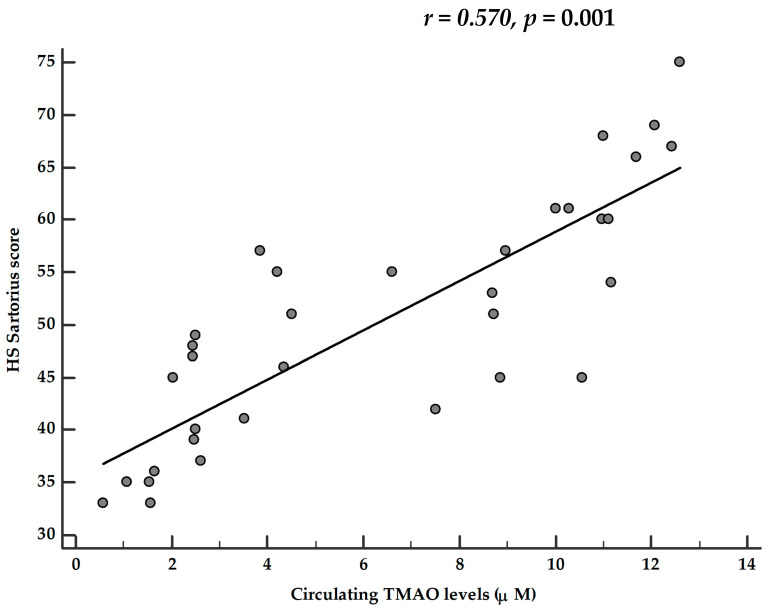
Partial correlation between HS Sartorius score and the circulating TMAO levels. HS Sartorius score was positively associated with the circulating TMAO levels, after adjusting for BMI, waist circumference, PREDIMED score, total energy intake, and PhA. Correlations between variables were performed using Pearson *r* correlation coefficients. A *p* value in bold type denotes a significant difference (*p* < 0.05). HS, Hidradenitis Suppurativa; TMAO, trimethylamine n-oxide.

The correlation between HS Sartorius score and the circulating TMAO levels (r = 0.570, *p* = 0.001) was still evident even after adjustment for confounding covariates, including BMI, waist circumference, PREDIMED score, total energy intake, and PhA (Figure 3).

Associations of the Hurley system with age, BMI, waist circumference, circulating TMAO levels, PREDIMED score, total energy intake, and PhA were assessed by a bivariate proportional OR model and are reported in Table 4. The Hurley system was significantly associated with all parameters, except for age (*p* = 0.650).

A linear regression model was performed to evaluate the association between HS Sartorius score (as a dependent variable) and BMI, waist circumference, circulating TMAO levels, PREDIMED score, total energy intake, and PhA as independent variables. Using this model, the circulating TMAO levels and PhA were the main predictors of the clinical severity of HS. Results are reported in Table 5.

## 4. Discussion

In this case-control study, we evaluated the circulating TMAO levels, a gut-derived metabolite associated with inflammation and cardiometabolic risk, in patients with HS, a highly disabling inflammatory skin disease associated with elevated prevalence of cardiometabolic comorbidities.

Our data demonstrated that the circulating TMAO levels were higher in HS patients than in control subjects. Consistently, we confirmed that patients with HS showed significant differences in PhA, indicative of an inflammatory status, and exhibited a lower adherence to the MD than controls. Of interest, the circulating TMAO levels were also associated with the clinical severity of the disease and this association was still evident after adjusting for common confounding covariates. Finally, the circulating TMAO levels and PhA were the main predictors of the clinical severity of HS. To the best of our knowledge, this is the first study that reported in HS patients a positive correlation between the circulating TMAO levels with the clinical severity of the disease.

TMAO is one of the most intensively studied gut-microbiome-derived metabolites in recent years [68] due to its association with an increased cardiometabolic risk [1]. TMA is produced by the gut microbiota from dietary carnitine, choline, and lecithin, and TMAO is the product of oxygenation of TMA in the liver by flavin-containing monooxygenase 3. It has previously been reported that increased circulating TMAO levels are a biomarker of an increased intestinal translocation of TMA in the presence of an altered intestinal barrier [2]. Several studies have previously revealed that high circulating TMAO levels were linked to several HS cardiometabolic comorbidities, including metabolic syndrome, insulin resistance, obesity, hypertension, and nonalcoholic fatty liver disease [69,70,71,72,73]. In addition, several studies have previously revealed increased intestinal permeability in patients with HS [19,20]. However, the association between HS and increased circulating TMAO levels was still unexplored. In line with our findings, Sikora et al. [74] very recently reported that in psoriasis, another chronic, immune-mediated disease of the skin associated with multiple systemic effects, increased circulating TMAO levels were associated with an altered gut barrier, gastrointestinal symptoms, and a systemic inflammatory profile. Of interest, TMAO has been demonstrated to activate pro-inflammatory signaling pathways [3]. Specifically, at physiologically circulating levels, TMAO binds to the endoplasmic reticulum stress kinase, thus inducing the forehead transcription factor FoxO1, a key driver of metabolic disease [75]. In that, it is tempting to speculate that, besides psoriasis, also in HS the increase circulating TMAO levels might represent the mechanistic link among diet, gut dysbiosis, and the inflammatory status, which in turn influence the disease severity and, possibly, the development of HS comorbidities. In support of this hypothesis, in our study the circulating TMAO level was one of the predictors of the clinical severity of HS, together with PhA, which has recently been proposed as a marker of inflammation [76].

We are aware that there are some limitations to the current study. First, the cause-effect association between the circulating TMAO levels and the clinical severity of HS cannot be determined due to the cross-sectional design of this study. Likewise, we cannot draw any final conclusions on the role of TMAO in the prediction of the clinical severity of HS. Second, the sample size is relatively small. Nevertheless, the sample size was calculated by using 70% statistical power that assured an adequate power to detect statistical significance. Third, the possible underlying inflammatory pathway linking circulating TMAO levels with the clinical severity of HS should be further investigated by measuring gut microbiota biomarkers, and the possible beneficial effects of the reduction of the circulating TMAO levels remains to be proven. Nevertheless, we point out that this was the first evidence reporting a positive correlation between the circulating TMAO levels and the clinical severity of HS, but future clinical studies are required to better clarify the role of TMAO in HS pathogenesis.

A major strength of this study was, however, the accurate characterization of the study population by a trained team of nutritionists, endocrinologists, and dermatologists. In particular, the diagnosis of HS was not self-reported, but clinically evaluated by two independent dermatologists, who were blinded to the design of the study, and patients included were only naïve to treatment. In addition, HS patients and controls were recruited with stringent exclusion criteria and were matched for sex, age, and BMI. Although the selection bias due to the single-center study design limits the generalizability of our findings, this design allowed us to increase the homogeneity of the sample. In fact, both HS patients and controls were recruited from the same geographical area, thus possibly sharing overall similar eating habits and food availability, as evidenced by the lack of difference in the adherence to the Mediterranean diet between the two groups. In addition, to further minimize the inter-operator variability, a single certified clinical nutritionist evaluated the nutritional status of both patients and controls and performed and interpreted BIA-parameters. In particular, the total energy intake was adequately assessed using the gold standard among food frequency questionnaires, the seven-day food record [77]. In addition, the PREDIMED questionnaire, which has also recently been validated in different Mediterranean countries including Italy [78], was not self-reported, but face-to-face administered, to reduce any bias related to the filling in of the questionnaire.

## 5. Conclusions

In summary, the results of the current study:

(i) Provide the first evidence that circulating TMAO levels were increased in HS patients and were associated with the clinical severity of the disease;
(ii) Improve the understanding of the association among diet, gut dysbiosis and inflammatory status in HS pathogenesis;
(iii) Support the detection of the circulating TMAO levels as an auxiliary assessment contributing to identifying HS patients who could get additional benefit from careful dietary interventions;
(iv) Suggest that the reduction of the circulating TMAO levels, through the indirect modulation of the gut microbiota and the enhancement of the intestinal barrier, could represent a potential important target in reducing the clinical severity of HS and the associated cardiovascular risk.

Finally, growing cooperation between nutritionists, endocrinologists, and dermatologist might be a promising combination in the complex management of HS patients.

## Figures and Tables

**Table 1:** Demographic information, anthropometric measurements, nutritional assessments, and BIA parameters of patients with HS and control group.

Parameters	HS Patients *n*. (%) or Mean ± SD *n*. 35	Controls *n*. (%) or Mean ± SD *n*. 35	*p*-Value
Sex, females (*n*, %)	24 (68.6%)	24 (68.6%)	χ^2^ = 0.07, *p* = 0.796
Age (years)	25.37 ± 8.36	26.14 ± 7.28	0.350
Smoking habit (yes)	18 (51.4%)	17 (48.6%)	χ^2^ = 0.00, *p* = 1.00
Physically active subjects	15 (42.9%)	13 (37.1%)	χ^2^ = 0.06, *p* = 0.807
BMI (kg/m^2^)	29.26 ± 5.33	29.22 ± 5.62	0.950
Normal-weight (*n*, %)	7 (20.0%)	10 (28.6%)	χ^2^ = 0.24, *p* = 0.624
Overweight (*n*, %)	12 (34.3%)	11 (31.4%)	χ^2^ = 0.00, *p* = 1.00
Grade I obesity (*n*, %)	12 (34.3%)	8 (22.9%)	χ^2^ = 0.63, *p* = 0.427
Grade II obesity (*n*, %)	4 (11.4%)	6 (17.1%)	χ^2^ = 0.12, *p* = 0.732
Waist circumference (cm)	92.19 ± 15.19	91.82 ± 15.51	0.833
PREDIMED score	7.68 ± 3.18	8.60 ± 2.31	**0.002**
PREDIMED categories			
Low adherence (*n*, %)	10 (28.6%)	3 (8.6%)	χ^2^ = 3.40, *p* = 0.065
Average adherence (*n*, %)	16 (45.7%)	20 (57.1%)	χ^2^ = 0.52, p = 0.473
High adherence (*n*, %)	9 (25.7%)	12 (34.3%)	χ^2^ = 0.27, *p* = 0.602
Total energy intake (kcal)	2233.30 ± 239.57	2271.22 ± 158.14	0.193
BIA parameters			
R (Ω)	492.77 ± 76.86	500.60 ± 66.17	0.695
Xc (Ω)	53.26 ± 9.37	59.37 ± 7.81	**0.010**
PhA (°)	6.18 ± 0.63	6.79 ± 0.60	**<0.001**

Differences between the two groups were analyzed by Student’s paired *t*-test. The chi square (χ^2^) test was used to determine the significance of differences in frequency distribution in categorical variables. Results were expressed as number (%) or mean ± SD. A *p* value in bold type denotes a significant difference (*p* < 0.05). HS, hidradenitis suppurativa; BMI, body mass index; PREDIMED, PREvención con DIeta MEDiterránea; BIA, bioelectrical impedance analysis; R, resistance; Xc, reactance; PhA, phase angle; SD, standard deviation.

**Table 2:** Differences in the Hurley system in demographic characteristics, anthropometric measurements, nutritional assessments, and BIA parameters in HS patients.

Parameters	HS Grade 1 *n*. (%) or Mean ± SD *n*. 12	HS Grade 2 *n*. (%) or Mean ± SD *n*. 23	*p*-Value
Sex, females (*n*, %)	8 (66.7%)	16 (69.6%)	χ^2^ = 3.11, *p* = 0.078
Age (years)	26.25 ± 8.89	24.91 ± 8.23	0.660
Smoking habit (yes)	2 (16.7%)	16 (69.6%)	χ^2^ = 12.63, ***p* = 0.001**
Physically active subjects	10 (83.3%)	5 (21.7%)	χ^2^ = 1.36, *p* = 0.244
BMI (kg/m^2^)	25.98 ± 4.61	30.97 ± 4.93	**0.007**
Normal-weight (*n*, %)	5 (41.7%)	2 (8.7%)	χ^2^ = 0.64, *p* = 0.426
Overweight (*n*, %)	4 (33.3%)	8 (34.8%)	χ^2^ = 0.91, *p* = 0.341
Grade I obesity (*n*, %)	3 (25.0%)	9 (39.1%)	χ^2^ = 2.51, *p* = 0.113
Grade II obesity (*n*, %)	0 (0%)	4 (17.4%)	χ^2^ = 2.39, *p* = 0.122
Waist circumference (cm)	83.80 ± 16.05	96.57 ± 13.03	**0.016**
PREDIMED score	9.83 ± 3.48	6.57 ± 2.41	**0.003**
PREDIMED categories			
Low adherence (*n*, %)	2 (16.7%)	8 (34.8%)	χ^2^ = 2.92, *p* = 0.087
Average adherence (*n*, %)	3 (25.0%)	13 (56.5%)	χ^2^ = 6.56, ***p* = 0.010**
High adherence (*n*, %)	7 (58.3%)	2 (8.7%)	χ^2^ = 2.04, *p* = 0.153
Total energy intake (kcal)	2093.67 ± 168.65	2306.17 ± 241.43	**0.005**
BIA parameters			
R (Ω)	508.50 ± 99.99	484.57 ± 62.65	0.390
Xc (Ω)	53.92 ± 11.68	52.91 ± 8.20	0.769
PhA (°)	6.73 ± 0.60	5.89 ± 0.42	**<0.001**

The chi square (χ^2^) test was used to determine the significance of differences in frequency distribution in categorical variables. Differences between the two groups were analyzed by Student’s impaired *t*-test. Results were expressed as number (%) or mean ± SD. A *p* value in bold type denotes a significant difference (*p* < 0.05). HS, hidradenitis suppurativa; BMI, body mass index; PREDIMED, PREvención con DIeta MEDiterránea; BIA, bioelectrical impedance analysis; R, resistance; Xc, reactance; PhA, phase angle; SD, standard deviation.

**Table 3:** Correlations between the clinical severity of HS with demographic characteristics, anthropometric measurements, nutritional assessments, and BIA parameters in patients with HS.

Parameters	HS Sartorius Score	*p*-Value
	*r*	
Age (years)	0.140	0.423
BMI (kg/m^2^)	0.443	**0.008**
Waist circumference (cm)	0.462	**0.005**
Circulating TMAO levels (µM)	0.840	**<0.001**
PREDIMED score	−0.538	**0.001**
Total energy intake (kcal)	0.403	**0.016**
R (Ω)	−0.342	**0.044**
Xc (Ω)	−0.105	0.549
PhA (°)	−0.857	**<0.001**

Correlations between variables were performed using Pearson *r* correlation coefficients. A *p* value in bold type denotes a significant difference (*p* < 0.05). HS, hidradenitis suppurativa; BMI, body mass index; PREDIMED, PREvención con DIeta MEDiterránea; BIA, bioelectrical impedance analysis; R, resistance; Xc, reactance; PhA, phase angle.

**Table 4:** Bivariate proportional odds ratio model to assess the association between Hurley system and age, BMI, waist circumference, circulating TMAO levels, PREDIMED score, total energy intake, and PhA.

Parameters	Hurley System			
	OR	*p-* Value	95% IC	R^2^
Age (years)	0.98	0.650	0.90–1.07	0.01
BMI (kg/m^2^)	1.24	**0.015**	1.04–1.48	0.20
Waist circumference (cm)	1.07	**0.026**	1.01–1.13	0.16
Circulating TMAO levels (µM)	2.16	**0.014**	1.17–3.99	0.48
PREDIMED score	0.70	**0.008**	0.53–0.91	0.22
Total energy intake (kcal)	1.00	**0.025**	1.00–1.01	0.19
PhA (°)	0.03	**0.004**	0.01–0.33	0.39

Bivariate proportional OR model, 95% IC, and R^2^. A *p* value in bold type denotes a significant difference (*p* < 0.05). HS, hidradenitis suppurativa; BMI, body mass index; TMAO, trimethylamine n-oxide; PREDIMED, PREvención con DIeta MEDiterránea; PhA, phase angle; OR, Odds Ratio; IC, Interval Confidence; R^2^, regression coefficient.

**Table 5:** Linear regression model of the association between HS Sartorius score (as a dependent variable) and BMI, waist circumference, circulating TMAO levels, PREDIMED score, total energy intake, and PhA as independent variables.

Parameters	Linear Regression Model							
	Non-Standardized Coefficients		Standardized Coefficients				Collinearity Statistics	
	T	SE	β	t	*p*-Value	95% IC	Tolerance	VIF
Circulating TMAO levels (µM)	1.520	0.404	0.545	3.76	0.001	0.69–2.35	0.26	3.92
PhA (°)	−7.864	2.396	−0.423	−3.28	0.003	−12.77–−2.96	0.32	3.11
PREDIMED score	−0.838	0.664	−0.229	−1.26	0.217	−2.19–0.52	0.16	6.16
BMI (kg/m^2^)	−0.481	0.401	−0.220	−1.20	0.240	−1.30–0.34	0.16	6.27
Waist circumference (cm)	−0.002	0.125	−0.003	−0.02	0.986	−0.26–0.25	0.20	4.93

Linear regression model. TMAO, trimethylamine n-oxide; PhA, phase angle; PREDIMED, PREvención con DIeta MEDiterránea; BMI, body mass index; T, statistic coefficient; SE, standard error; IC, interval confidence; VIF, variance inflation factor.

## Data Availability

Results attained in this study are included in the manuscript. Individual data are not publicly available due to ethical restrictions.

## Abbreviations

HS: Hidradenitis Suppurativa; MD, Mediterranea Diet; BMI, body mass index; PhA, phase angle; BIA, bioelectrical impedance analysis; TMAO, Trimethyilamine *N*-oxide; SD, standard deviation; PREDIMED, Prevención con Dieta Mediterránea; Xc, reactance; R, resistance; OR, Proportional Odds Ratio; IC, Interval Confidence.
